# Psychological distress among greek parents of children with an autism spectrum disorder: Is there a link to anxiety symptoms in neurotypical offspring?

**DOI:** 10.1192/j.eurpsy.2021.1635

**Published:** 2021-08-13

**Authors:** E. Koukouriki

**Affiliations:** 1 Special Education Lab, Department Of Primary Education, University of Ioannina, Ioannina, Greece; 2 Centre For Educational And Counseling Services Of Trikala, Hellenic Ministry of Education and Religious Affairs, Trikala, Greece

**Keywords:** psychological distress, anxiety symptoms, autism spectrum disorder, neurotypical offspring

## Abstract

**Introduction:**

Parents of children with an autism spectrum disorder (ASD) are at higher risk of suffering from mental health problems (MHP) than parents of children with other developmental disabilities. Research on the general population documented that MHP in parents contributes as a significant risk factor for maladjustment outcomes in their offspring, while parental anxiety disorders in particular have been associated with a higher prevalence of anxiety disorders in the offspring. However, evidence concerning possible associations between parent-offspring psychological problems in the case of ASD-families is scarce and inconsistent.This study forms a part of a larger PhD study and some preliminary findings have been partially discussed in previous work.

**Objectives:**

To investigate any association between anxiety symptoms in neurotypical offspring (ASD-siblings) with parental MHP.

**Methods:**

118 parent-child-dyads from ASD-families that fulfilled inclusion criteria participated in this study and answered a demographic questionnaire. Parents were administered the GHQ-28, while the children answered the STAIC-A-Trait. A hierarchical multiple regression was performed to test the hypothesis.

**Results:**

Participating parents demonstrating poorer mental health, with 53 (44.9%) of them considered as suffering from psychological distress according to the score of GHQ-28. Furthermore, hierarchical regression showed that STAIC-(A-Trait) total score of ASD-siblings was associated with parental anxiety (std beta=0.292; p=0.29;model 1) and this association persisted after demographics entered the model (std beta=0.300; p=0.029; model 2).

**Conclusions:**

This study shows a significant association between parental psychological distress and anxiety in the neurotypical offspring in ASD-families, highlighting the need to provide all members of ASD-families with the appropriate services.

**Disclosure:**

No significant relationships.

